# Microbiota-Derived Propionate Modulates Megakaryopoiesis and Platelet Function

**DOI:** 10.3389/fimmu.2022.908174

**Published:** 2022-07-08

**Authors:** Kerstin Dürholz, Eva Schmid, Michael Frech, Vugar Azizov, Nadine Otterbein, Sébastien Lucas, Manfred Rauh, Georg Schett, Heiko Bruns, Mario M. Zaiss

**Affiliations:** ^1^ Department of Internal Medicine 3, Rheumatology and Immunology, Friedrich-Alexander-University Erlangen-Nürnberg (FAU) and Universitätsklinikum Erlangen, Erlangen, Germany; ^2^ Deutsches Zentrum für Immuntherapie (DZI), Erlangen, Germany; ^3^ Department of Allergy and Pneumology, Children’s Hospital, Friedrich-Alexander-Universität (FAU) Erlangen-Nürnberg, Universitätsklinikum Erlangen, Erlangen, Germany; ^4^ Department of Internal Medicine 5, University Hospital Erlangen, Erlangen, Germany

**Keywords:** rheumatoid arthritis, microbiota, short-chain fatty acids, hematopoietic progenitors, megakaryocytes, platelets

## Abstract

Rheumatoid arthritis (RA) is associated with an increased risk for cardiovascular events driven by abnormal platelet clotting effects. Platelets are produced by megakaryocytes, deriving from megakaryocyte erythrocyte progenitors (MEP) in the bone marrow. Increased megakaryocyte expansion across common autoimmune diseases was shown for RA, systemic lupus erythematosus (SLE) and primary Sjögren’s syndrome (pSS). In this context, we evaluated the role of the microbial-derived short chain fatty acid (SCFA) propionate on hematopoietic progenitors in the collagen induced inflammatory arthritis model (CIA) as we recently showed attenuating effects of preventive propionate treatment on CIA severity. *In vivo*, propionate treatment starting 21 days post immunization (dpi) reduced the frequency of MEPs in the bone marrow of CIA and naïve mice. Megakaryocytes numbers were reduced but increased the expression of the maturation marker CD61. Consistent with this, functional analysis of platelets showed an upregulated reactivity state following propionate-treatment. This was confirmed by elevated histone 3 acetylation and propionylation as well as by RNAseq analysis in Meg-01 cells. Taken together, we identified a novel nutritional axis that skews platelet formation and function.

## Introduction

Short chain fatty acids (SCFA) are bacterial metabolites produced in the colon utilizing for the host indigestible fibers (1). Interestingly, the carbon 3 SCFA propionate (C3) shows effects on a variety of body compartments and cells, including the hematopoietic progenitor niche in the bone marrow (2). As such, C3 was shown to affect disease outcome in mouse models of allergic airway disease, acute radiation syndrome as well as osteoporosis (3–5). Interestingly, hematopoietic progenitor cells from patients with rheumatoid arthritis (RA) show a phenotype characterized by defective proliferative capacity (6). RA is a chronic and frequently progressive disease which is characterized by a persistent inflammation of synovial joints (7). In mice with collagen induced arthritis (CIA) - a preclinical RA model - the disease led to an expansion of myeloid cells (8). In addition to the joint pathology, RA patients suffer from pro-inflammatory processes on a systemic level. Generally, under inflammatory conditions, the frequency and ratio of the different hematopoietic progenitor populations changes (6, 8, 9). These changes in hematopoietic progenitor populations during inflammation go together with changes in blood parameters including platelet count and function as well as the risk for thrombosis (10, 11). As shown in a study conducted on RA patients with active inflammatory arthritis, and RA patients in a non-active disease state, a significant higher platelet reactivity was observed in patients with active disease (12). Moreover, also the number, distribution and the volumetric indices of platelets were shown to be altered in RA patients (13–16). Notably, studies reveal an increased risk for RA patients for cardiovascular mortality (17, 18), which negatively correlates with dietary fiber intake (19). Moreover, platelet parameters can be shaped by dietary patterns (20). In this context we evaluated the role of the SCFA C3 on hematopoietic progenitor cells in the CIA mouse model for RA as well as under steady state conditions.

## Methods

### Mice and Treatments

Five to six week old WT C57BL/6N (Charles River) and DBA/1J mice (Janvier) were acclimated for 1 week, followed by a 3 week co-housing period before starting the experiments. All mice were maintained under specific pathogen-free conditions at the Präklinisches Experimentelles Tierzentrum (PETZ) Erlangen, Germany and approved by the local ethics authorities of the Regierung of Unterfranken (#55.2-2532-2-424). Supplementation of sodium propionate (Sigma-Aldrich) was done in the drinking water at a final concentration of 150 mM and changed every 3 days. The animals received water w/wo C3 and standard chow (Ssniff Spezialdiäten GmbH) ad libitum.

### Collagen-Induced Arthritis

CIA was induced in 8-week-old female DBA/1J mice by subcutaneous injection at the base of the tail with 100 µl of 0.25 mg chicken type II collagen (CII; Chondrex) in complete Freund adjuvant (CFA; Difco Laboratory) containing 5 mg/ml killed Mycobacterium tuberculosis (H37Ra). Mice were re-challenged after 21 days intradermal immunization in the base of the tail with this emulsion. The paws were evaluated for joint swelling three times per week. Each paw was individually sored using a 4-point scale: 0, normal paw; 1, minimal swelling or redness; 2, redness and swelling involving the entire forepaw; 3, redness and swelling involving the entire limp; 4, joint deformity or ankylosis or both.

### Measurement of Serum Cytokines

Serum cytokines were measured with the LEGENDplex™ MU Th Cytokine Panel (Biolegend) following the manufacturer’s instructions.

### Histology

For histological analysis, paws were fixed in 4% formalin for 12 h and decalcified in EDTA (Sigma-Aldrich). Serial paraffin sections (2 µm) were stained for H&E.

### Flow Cytometry

Bone marrow was flushed out of femurs with PBS using a 27 G needle and smashed through a 70 µM cell strainer. Single-cell suspensions were then stained for flow cytometry with the following antibodies: Lineage-Cocktail (BV421), CD34 (FITC), CD117 (PE), CD127 (APC), Ly6A/E (PE/Cy7), CD16/32 (APC/Cy7), CD45 (AF700), CD61 (PE), CD41 (FITC). Bone marrow cells were gated as previously described by Luo et al. (2015) (21). Meg-01 cells were collected, washed with PBS and then stained with CD61 (PE) and CD41 (FITC). For the flow cytometric analysis of megakaryocytes, cells were gated as described by Matsumura-Takeda et al. (2007) with some modifications (22).

### Platelet Aggregation Analysis

The effect of C3 on the platelet aggregation potential was assessed using a Multiplate^®^ (Dynabyte) platelet function analysis (23). Therefore, blood was taken from C3 treated and control mice *via* cardiac puncture with a 27 G needle, and directly transferred with a Hirudin coated syringe to avoid clotting. The samples were incubated at least 30 minutes at RT before the analysis. Collagen (100 µg/ml) and Adenosine diphosphate (ADP) (0.2 mM) were used as agonists for platelet aggregation as previously described (24–26).

### *In Vitro* Experiments and RNA Sequencing With the Meg-01 Cell Line

Meg-01 cells were purchased from the DSMZ - German Collection of Microorganisms and Cell cultures GmbH (ACC364) and cultured in RPMI Medium supplemented with 10% FCS, 1% Penicillin-Streptavidin and 2mM glutamine in a humidified incubator at 37°C, 5% CO2. For experiments, cells were seeded at a concentration of 0.3 x 10^6^ cells/ml. For RNA extraction cells were incubated for 24 h and 48 h in a humidified incubator at 37°C, 5% CO2 until RNA extraction. For monitoring of megakaryocyte maturation cells were grown in medium containing 250 µM C3 or 1 nM phorbol 12-myristate 13-acetate (PMA) as a positive control and incubated for 7, 14 or 21 days until flow cytometric analysis. For the assessment of histone acetylation *via* western blotting, Meg-01 were stimulated with 500 µM C3 for 48 hours. Cells grown without C3 served as negative controls. The Illumina RNA sequencing (RNAseq) analysis was performed by Novogene Sequencing – Europe (UK, Cambridge Sequencing Center). In brief, sequencing libraries were generated using NEBNext^®^ Ultra TM RNA Library Prep Kit for Illumina^®^ (NEB, USA) and sequenced on an Illumina platform. Raw data (raw reads) of FASTQ format were processed through fastp. Mapping of the processed data to the reference genome homo sapiens (GRCh38/hg38) was performed using the Spliced Transcripts Alignment to a Reference (STAR) software (27). FeatureCounts was used for the quantification of the mapped reads (28). Raw mapped reads were processed in R (Lucent Technologies) with DESeq2 (29), to determine differentially expressed genes and generate normalized read counts. Pathway enrichment analysis was performed using the free online platform DAVID (30).

### Quantitative PCR

RNA was isolated using Genazol (Genaxxon) following manufacturer’s instructions. Gene expression results are expressed as arbitrary units relative to expression of the house keeping gene GAPDH. Primer sequences are as follows:

GAPDH: 5’-TGATGACATCAAGAAGGTGGTGAAG-3’ and 5’-TCCTTGGAGGCCATGTGGGCCAT-3’; CD61: 5′-AGGCCCTCGAAAACCCCTGCT-3′ and 5′- GCCACCCTCTGGGGCATCTC -3′

### Histone Extraction and Western Blotting

Meg-01 cells were pre-treated with 500 µM C3 and only medium as a negative control for 48 hours. Cells were then washed with ice cold PBS supplemented with 5 mM sodium butyrate. Then the cytoplasm was extracted by resuspending cells in ice cold extraction buffer at 10^7^ cell/mL, incubating on ice for 5 minutes, and centrifuging at 6500xg for 10 minutes at 4°C. Supernatant was discarded and the cytoplasm extracted for the second time. Histones were extracted by resuspending the pellet in 0.25 M HCl at a density of 4x10^7^ cells/mL. Samples were then sonicated for 30 seconds, placed on ice, then sonicated for further 30 seconds. Afterwards, tubes were placed on rollers at 4°C for 1 hour. Then, samples were centrifuged at 12000xg for 10 minutes at 4°C. Supernatant was collected and neutralized with 2M NaOH at 1/10th of the volume of the supernatant. Extracted histones were frozen and stored at -80°C until analyzed by western blotting. Protein extracts were separated on 4-12% Bis-Tris gradient SDS-polyacrylamide gel, then transferred onto PVDF membrane, blocked with 5% milk in TBS 0.05% Tween 20 for 1 hour. To assess Histone 3 propionylation levels membranes were probed with anti-propionyl-histone H3 (lys23) Mouse mAB (# PTM-208, PTM BIO). For determination of Histone acetylation levels membranes were probed with anti-acetyl-lysine-27-histone-3 (ab 4729, abcam). For normalization membranes were probed with an anti-histone 3 antibody (9715S, cell signaling). For visualization appropriate HRP-conjugated secondary antibodies were used (donkey anti rabbit (ab6802, abcam); m-IgG FC BP-HRP (sc-525416, santa cruz). The signal acquired from chemiluminescence (Celvin S) for protein of interests were optimized to loading control and quantified with Image J blot analysis tool.

### Statistical Analysis

Statistical analyses were performed using Prism 8 software (GraphPad). Comparisons between two groups were performed using unpaired or paired, two-tailed, Student’s t test. Comparisons between more than two groups were performed using one-way ANOVA and Tukey’s or Dunnett’s multiple comparison test. Statistical significance: * p < 0.05; ** p < 0.01; *** p < 0.001; **** p < 0.0001. Details on the statistical analysis are listed in the figure legends.

## Results

### Propionate Alleviates CIA Symptoms and Alters Bone Marrow Progenitor Distribution

**Figure 1:**
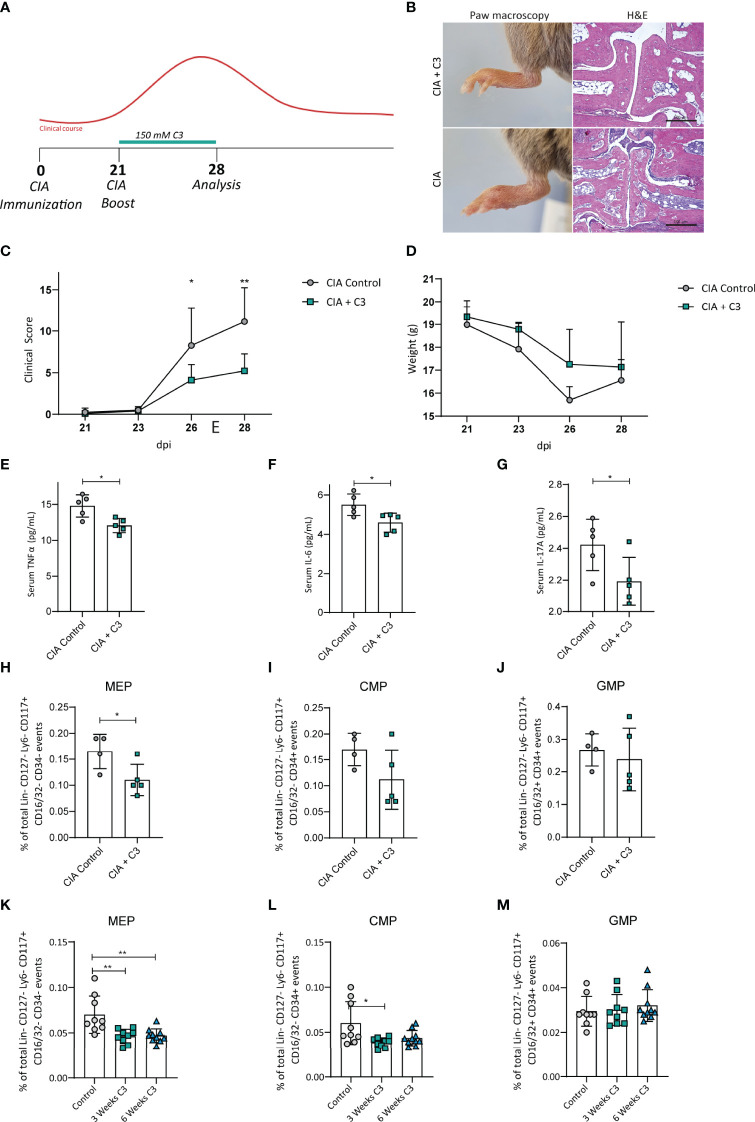
C3 treatment induces changes of bone marrow progenitor cells in mice with CIA and naïve WT mice. **(A)** Flow diagram illustrating the experimental setup of C3 treatment **(B)** H&E staining of paw sections, **(C)** joint swelling clinical scores and **(D)** weight of CIA controls and CIA treated with 150 mM C3 in the drinking water. **(E)** Serum cytokine levels of TNFα **(F)** IL-6 and **(G)** IL-17A of CIA controls and CIA treated with 150 mM C3 in the drinking water. **(H)** Total percentage of bone marrow MEP, **(I)** CMP and **(J)** GMP of CIA controls and CIA treated with 150 mM C3 in the drinking water. **(K)** Total percentage of MEP, **(L)** CMP and **(M)** GMP in untreated naïve controls and naïve mice treated with 150 mM C3 in the drinking water for 3 and 6 weeks. Pictures are representative for 2 independent experiments. Data are expressed as the mean ± sd. Statistical difference was determined by Two-way ANOVA, Student’s t-test and One-way ANOVA. *p < 0.05; **p < 0.01.

To determine the effect of C3 on arthritis progression and bone marrow progenitor populations in mice, we supplemented CIA mice with C3 with the onset of clinical arthritis scores starting at 21 days post immunization (dpi) in the drinking water (**Figure 1A**). Interestingly, beside our previously published results on C3 treatment throughout the experimental CIA model (4), we identified that nutritional supplementation with C3 was equally effective to promote significantly lower arthritis scores compared to untreated controls by starting at 21 dpi (**Figures 1C, D**). Furthermore, H&E staining of paw sections showed a prominent decrease in leukocyte infiltration in C3 treated mice compared to the control group (**Figure 1B**). The measurement of cytokines by a Legendplex assay revealed significant lower levels of TNFα (**Figure 1E**), IL-6 (**Figure 1F**) and IL-17A (**Figure 1G**) in the serum after C3 treatment. Analysis of bone marrow progenitor populations at day 28 post CII immunization revealed a significant decrease in the MEP population (**Figure 1H**), whereas CMP and GMP percentages remained unchanged (**Figures 1I, J**). To assess whether this effect is disease-dependent or a general effect of C3, we treated naïve WT mice for 3 to 6 weeks with C3 in the drinking water. Consistent with the results in the CIA model, we observed a significant decrease in MEP after three and six weeks following C3-treatment (**Figure 1K**). Of note, GMP populations again remained unchanged (**Figure 1M**) whereas after 3 weeks treatment a significant decrease of CMP was observed (**Figure 1L**). As MEP are the precursors of erythrocytes and platelets we investigated if there are changes in standard platelet parameters such as platelet counts, volume and mean platelet component. However, blood analysis revealed no changes in erythrocyte or platelet-parameters following nutritional C3 supplementation in CIA and WT mice (**Figure S1**).

### Propionate Reduced Megakaryocyte Numbers but Promoted Maturation

**Figure 2:**
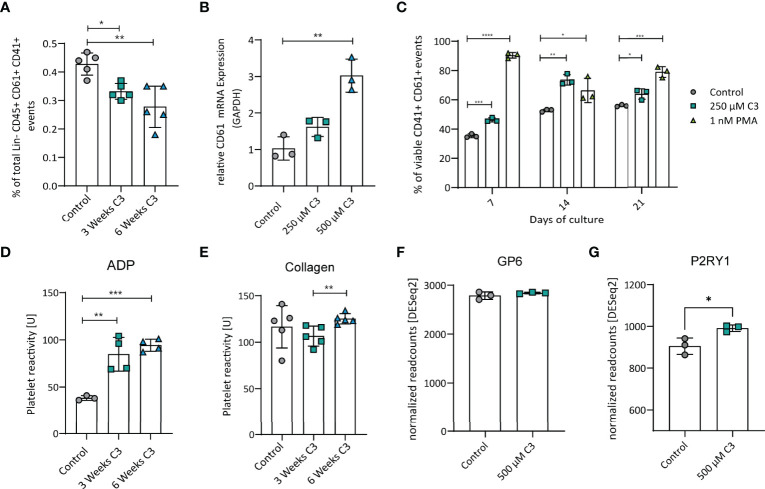
C3 reduced megakaryocyte numbers *in vivo* but promoted maturation *in vitro* and increased reactivity of platelets to ADP after three or six weeks of C3 treatment but not to collagen. **(A)** Total percentage of Megakaryocytes in the bone marrow after three and six weeks of C3 treatment. **(B)** Gene expression CD61 in Meg-01 cell line after treatment with 250 µM and 500 µM C3. **(C)** Percentage of viable CD41^+^ CD61^+^ Meg-01 after treatment with 250 µM C3 or 1 nM PMA as a positive control after 7, 14 or 21 days of treatment *in vitro*. **(D)** Platelet reactivity to ADP after three or six weeks of C3 treatment. **(E)** Platelet reactivity to collagen after three or six weeks of C3 treatment. **(F)** Gene expression of GP6 in Meg-01 cells between control and 500 µM C3 treatment. **(G)** Gene expression of P2RY1in Meg-01 cell between control and 500 µM C3 treatment. Data are expressed as the mean ± sd. Statistical difference was determined by Two-way ANOVA, Student’s t-test and One-way ANOVA. *p < 0.05; **p < 0.01; ***p < 0.001; **** means p < 0.0001.

MEP give rise to megakaryocytes that are in turn responsible to produce blood platelets. Therefore, we next analysed megakaryocytes after C3 treatment. Flow cytometric analysis of bone marrow cells revealed significantly decreased percentage of megakaryocytes after three or six weeks of C3 treatment (**Figure 2A**). *In vitro*, C3 treatment of the megakaryoblastic cell line Meg-01, upregulated the maturation marker CD61 on mRNA level (**Figure 2B** and **Figure S1**). In addition, flow cytometric analysis of Meg-01 after 7, 14 or 21 days of 250 µm C3 stimulation, confirmed increased surface expression of CD61 over untreated controls (**Figure 2C**). These data show that C3 treatment leads to a decrease in megakaryocytes frequencies *in vivo* along with an increased surface expression of the maturation marker CD61 *in vitro*.

### Propionate Selectively Increased Platelet Reactivity

Next, to address whether platelets from C3-treated mice have altered function compared to those from untreated controls, we isolated platelets from naïve WT mice after 3 or 6 weeks of C3 treatment. Coagulation activity was analysed using two standard-stimuli, adenosine diphosphate (ADP) and collagen, to induce platelet aggregation (26). Platelets from C3 treated mice showed increased reactivity towards ADP, whereas - compared to controls - no significant effect on collagen induced platelet reactivity was observed (**Figures 2D, E**). These findings match with results from our mRNA data in the Meg-01 cell line that shows megakaryocyte characteristics such as RNA content and surface receptors being shared between mother cell and produced platelets (31). The selective increase in reactivity towards ADP but not collagen could be explained by changes in receptor expression responsible for coagulation signalling upon a specific stimulus. After C3 treatment, Meg-01 cells expressed significantly higher levels of P2RY1 (32), the receptor responsible for reactivity towards ADP (**Figure 2G**), whereas GP6 expression, responsible for acting upon collagen stimulation (33), remained unaltered (**Figure 2F**) in the current setting. These results indicate a selective effect of C3 treatment on platelet reactivity towards ADP but not collagen possibly *via* alterations in surface receptor expression.

### Propionate Upregulates Megakaryocyte Differentiation and Platelet Activation Pathways

**Figure 3:**
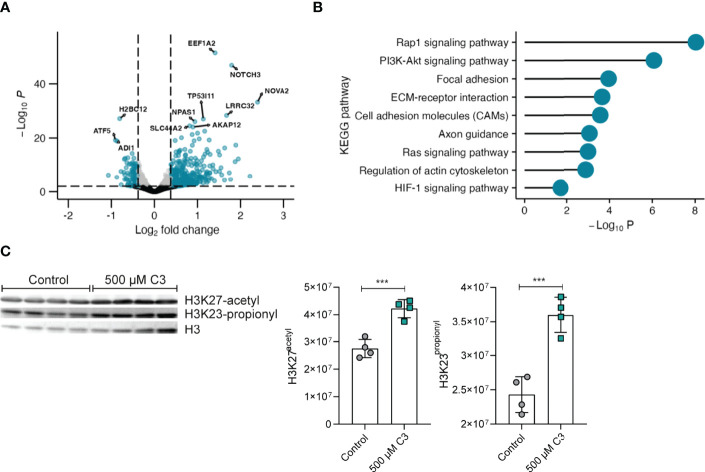
C3 significantly upregulates expression of genes involved in megakaryocyte maturation. **(A)** Volcano plot of fold change (FC) values of mRNA sequencing RNAseq analysis from Meg-01 cells treated with 500 μM C3 compared to non-treated cells (control). n = 3 technical replicates. Gates were set at an adjusted p-value < 0.01 and a fold change > 30% and depicted as log2 fold change. Genes marked in blue display genes above log2 fold change gate and p-value gate. Grey dots display genes below the log2 fold change gate and above the p-value gate. Black dots display genes below the log2 fold change and p-value gate. **(B)** KEGG pathway analysis of the upregulated differential expressed genes (DEGs) identified from comparison of Meg-01 cells cultured in 500 μM C3 vs. untreated cells (Control). DEGs were filtered on adjusted p-values lower than 0.01 and fold changes greater than 30%. **(C)** Western blot analysis of H3 histone acetylation and propionylation of Meg-01 after stimulation with 500 µM C3. Untreated cells (only medium) served as negative controls (n ≥ 3 technical replicates). Data are expressed as the mean ± sd. Statistical difference was determined by ordinary one-way ANOVA. ***p < 0.001.

To further investigate potential C3 effects on megakaryocytes in general we moved on with RNAseq of Meg-01 cells. Taking a closer look at the gene expression profile (Excel table with significant DEGs in the online **Supplementary Material**), C3 treatment induced a pronounced upregulation of genes as opposed to downregulation (**Figure 3A**). Highly upregulated genes (fold change < 30%, adjusted p-value < 0.01) include NOVA alternative splicing regulator 2 (NOVA2), notch receptor 3 (NOTCH3) and SLC44A2 - genes involved in cell fate decisions (34), megakaryocyte differentiation (35) and platelet activation (36). Of interest, RNAseq analysis confirmed identified upregulated genes such as CD61 in isolated bone marrow megakaryocytes following *in vivo* C3 treatment (**Figure S1**). To assess the biological meaning of differentially expressed genes (DEG) upon C3 treatment, we performed a KEGG pathway analysis of the DEG using the Functional Annotation Tool at the DAVID bioinformatics database (**Figure 3B**). DEGs were defined as genes with a fold change greater than 30% and an adjusted p-value < 0.01. DEGs upregulated upon C3 treatment are involved in Rap1 signalling, cell adhesion molecules as well as the regulation of the actin cytoskeleton – pathways associated with megakaryocyte maturation (37, 38). To test whether changes in histone acetylation or propionylation could be responsible for the observed modified transcriptional activation, we analysed histones for these post-translational modifications (PTMs) in Meg-01 cells. Immunoblotting for H3 in acidic histone extracts following C3 stimulation in Meg-01 cells revealed significantly increased acetylation (H3K27) and propionylation (H3K23) (**Figure 3C**). This is of great interest and will allow new experimental designs in follow-up studies to in depth asses the role of C3 in the context of cardiovascular events during RA.

## Discussion

In the current study, we found that C3 consumption significantly reduced the frequency of the MEP population in the bone marrow of CIA and naïve mice. Despite subsequent lower megakaryocyte numbers, no differences were observed in typical platelet parameters such as platelet counts, MPV and MCP values. However, our observed increased functional platelet reactivity assays along with C3 induced overall transcriptional activity of platelets and Meg-01 cells further emphasized previous findings showing that both megakaryocytes and platelets are sensible towards extrinsic influences and can adapt to conditions such as the nutritional state and inflammation (21, 39). Further, our findings are of high relevance in light of a recent publication providing clear evidence for peripheral megakaryocyte expansion across common autoimmune diseases such as RA, systemic lupus erythematosus (SLE) and primary Sjögren’s syndrome (pSS) (40). In peripheral blood, megakaryocytes are a heterogeneous cell population that comprises a subpopulation with distinct immune characteristics in these autoimmune diseases. The authors discuss the idea that that the observed megakaryocyte expansion might initially prime autoimmune T cells in the pathogenesis of these autoimmune diseases. Although very speculative at the current state, prophylactic C3 nutritional supplementation was shown to effectively attenuate inflammatory arthritis onset (4) and here we provide additional data showing that C3 treatment at the time of clinical onset is also effective in attenuating disease symptoms. Another recent study reported that supplementation with a high fibre-rich diet - providing the fuel for increased C3 levels by fermentation process of the gut microbiota - improved lupus-related disease manifestations in a Toll-like receptor 7-dependent lupus-like mouse model (41). Last, as for pSS the involvement of the gut microbiota along with SCFAs was well reviewed by Zhang et al. (42) this further suggested the general potential benefit of C3 supplementation on autoimmune diseases *via* modulation of megakaryocytes. A direct role of megakaryocytes in the susceptibility of arthritis was shown by a study on IgG-mediated arthritis in different mouse models of kit-insufficiency. Kit-insufficiency normally serves as a model for mast cell deficiency – however also megakaryocytes are affected – with either increased or decreased levels of megakaryocytes depending on the type of kit-deficiency model used. Interestingly, kit-insufficient mice with low levels of megakaryocytes are resistant to arthritis induced by transfer of IgG autoantibody–containing serum from K/BxN mice. Thereby, the transfer of megakaryocytes restored the susceptibility whereas mice which received IL-1 -/- megakaryocytes remained resistant to the disease. Mechanistically Il-1 was shown to activate synovial fibroblast which may be the link between megakaryocytes and inflammatory arthritis in this model (43).

Performing RNAseq analysis of the megakaryoblastic cell line Meg-01 we could show that C3 regulates gene-expression which may affect their functional role in the disease course of arthritis. The strongest upregulated genes upon C3 treatment in megakaryocytes include the NOVA alternative splicing regulator 2 (Nova2), and the notch receptor 3 (Notch3). Nova2 regulates pre-mRNA splicing and was shown to be involved in cell fate decisions for assignment of cell fate in the context of vascular differentiation (34). Notch 3 protein is a transmembrane receptor and was recently shown to be involved in late megakaryocyte differentiation (35). Transcriptomic changes might be linked to our here reported histone post-translational modifications, where histone acetylation and propionylation are marks of active chromatin (44–46). Further, flow cytometry analysis revealed that C3 also increased CD61 surface expression, a marker of megakaryocyte maturation. Thus, C3 seems to affect megakaryocyte maturation *in vitro*, although we see lower frequencies of megakaryocytes and their progenitors *in vivo*. The lower abundance of megakaryocytes *in vivo* – also shown in the arthritis resistant kit-insufficient mice (43)– could at least partly explain the lower arthritis scores during C3 treatment, maybe due to lower Il-1 dependent synovial fibroblast activation. However, here we do not provide data showing the direct link between C3 effects on megakaryocytes and their progenitors and arthritis symptoms, Therefore, identifying the effect of C3 on the whole differentiation progress from HSC to megakaryocyte using HSC tracing, as previously described by Grinenko et al. (47), would be an interesting approach. Moreover, the direct effect of C3 on the secretion of Il-1 by megakaryocytes under pro-inflammatory conditions, would be interesting to assess in an *in vitro* approach. As in RA frequencies of peripheral megakaryocytes are changed (48) it would be further worth investigating if the effect of C3 is also directed towards the peripheral population. Certainly, although further studies are needed here to unravel the consequences and identify potential therapeutic approaches, our here identified differences in platelet functionality and their progenitors support the concept that megakaryocytes effectively confer immune functions in RA and SLE (49) which are altered upon C3 treatment.

## Conclusions

Our findings identified a novel nutritional axis that skews platelet formation and function. This data could serve as a first step in identifying novel therapeutic approaches additionally to the preventive setting as recently shown by our group. Further, our data may suggest that C3 nutritional supplementation could directly support the reported inflammatory arthritis attenuating effects by modulating megakaryocyte numbers and platelet immunomodulatory functions.

## Limitations

Although the finding from this study has a high potential, especially in the line of recent findings on increased megakaryocyte numbers in autoimmune diseases, the data presented here as a brief research report are mainly descriptive and follow-up studies are needed to better interpret the results. Functional consequences of modulated platelet reactivity in inflammatory arthritis models and in depth ChIP analysis in megakaryocytes to link upregulated genes with increased histone acetylation and propionylation at the respective genomic loci would further strengthen our observations. Further, Mulan et al. identified a general platelet hyper-reactivity to ADP – as observed following C3-treatment in our study - in inflammatory arthritis and suggested this a new potential therapeutic target which maybe a critical point to mention (50).

## Data Availability Statement

The datasets presented in this study can be found in online repositories. The name of the repository and accession number can be found below: NCBI Gene Expression Omnibus; GSE202712.

## Ethics Statement

The animal study was reviewed and approved by Local ethics authorities of the Regierung of Unterfranken, Germany (#55.2-2532-2-424).

## Author Contributions

KD, ES performed most of the experiments. NO, SL, MR performed the Platelet Aggregation Analysis. VA performed Western blot experiments. MF performed and analysed bulk RNAseq. GS, HB and MZ designed the experimental work. MZ coordinated the study. KD, ES, and MZ wrote the article with inputs from co-authors. All authors contributed to the article and approved the submitted version.

## Funding

Funding: KD, ES, MZ are funded by the Deutsche Forschungsgemeinschaft (DFG- CRC1181 project B07), MF is funded by Deutsche Forschungsgemeinschaft (DFG-FOR2886 project A1). ES is further funded by the IZKF intramural funds of the medical faculty at the Friedrich-Alexander-Universität Erlangen-Nürnberg (FAU), Germany. GS is funded by the H2020 GA 810316 - 4D-Nanoscope ERC Synergy Project, the IMI funded projects RTCure and HIPPOCRATES, the Emerging Fields Initiative MIRACLE of the FAU. VA is funded by Deutsche Forschungsgemeinschaft (DFG-FOR2886 project A3.

## Conflict of Interest

The authors declare that the research was conducted in the absence of any commercial or financial relationships that could be construed as a potential conflict of interest.

## Publisher’s Note

All claims expressed in this article are solely those of the authors and do not necessarily represent those of their affiliated organizations, or those of the publisher, the editors and the reviewers. Any product that may be evaluated in this article, or claim that may be made by its manufacturer, is not guaranteed or endorsed by the publisher.

## Supplementary Material

Supplementary Figure 1 C3 does not alter platelet and erythrocyte parameters in whole blood from mice with CIA and naive WT mice. Platelet parameters include **(A)** Platelet count, **(B)** Mean platelet volume (MPV) and **(C)** Mean platelet component (MPC). Erythrocyte parameters include **(D)** Red blood cell (RBC) count, **(E)** Mean corpuscular volume (MCV) and **(F)** Mean corpuscular haemoglobin concentration (MCHC). **(G)** Gene expression of CD61 in Meg-01 from RNAseq Expressed as DESeq2 normalized readcounts. Data are expressed as the mean ± sd. Statistical difference was determined by Student’s t-test. **p < 0.01.
