# Supplementary figures and images for: Microbiota-Derived Propionate Modulates Megakaryopoiesis and Platelet Function
Source: Front Immunol. 2022 Jul 8;13:908174. doi: 10.3389/fimmu.2022.908174 (PMC9307893; doi:10.3389/fimmu.2022.908174)

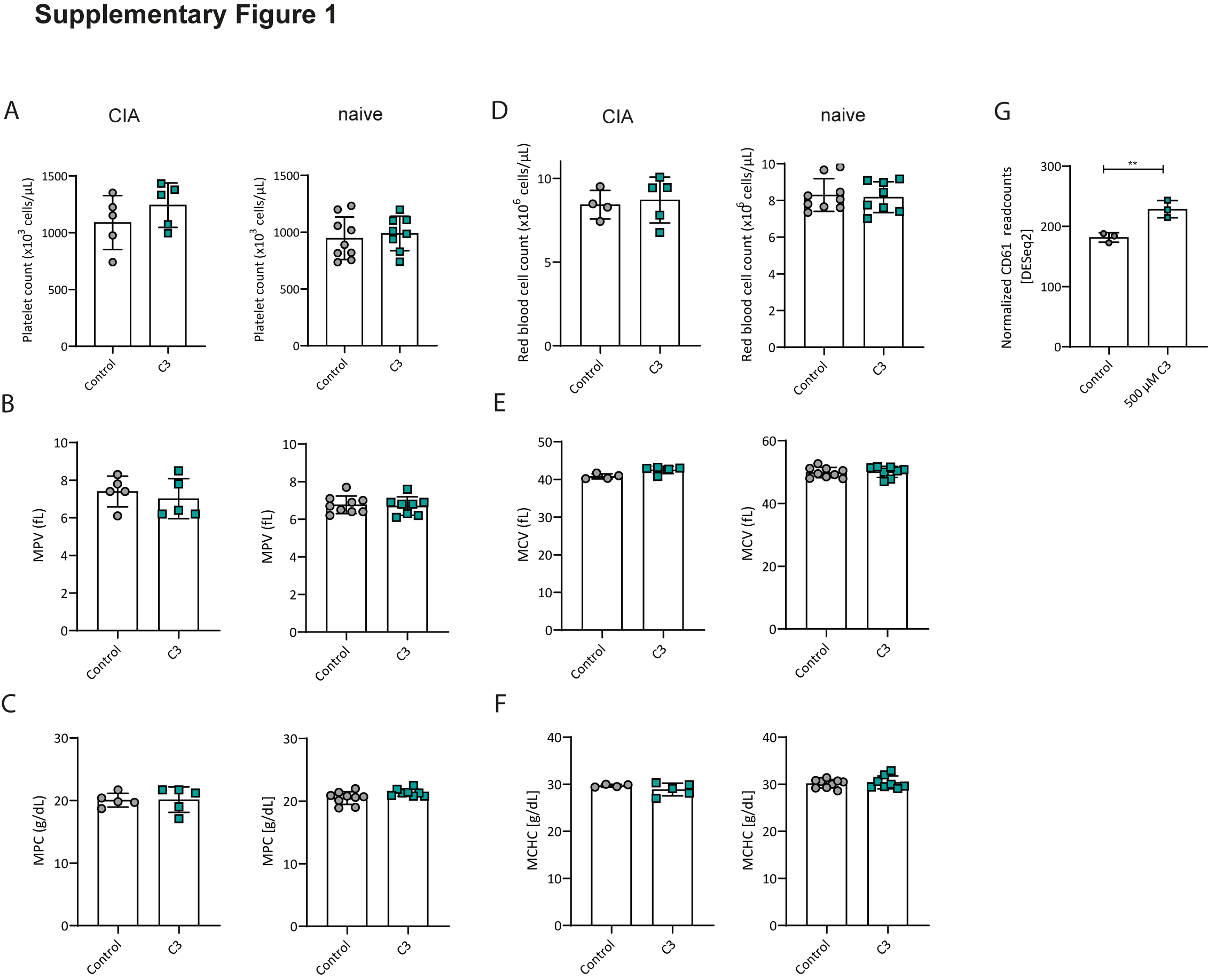

Supplement: Supplementary Figure 1 — C3 does not alter platelet and erythrocyte parameters in whole blood from mice with CIA and naive WT mice. Platelet parameters include (A) Platelet count, (B) Mean platelet volume (MPV) and (C) Mean platelet component (MPC). Erythrocyte parameters include (D) Red blood cell (RBC) count, (E) Mean corpuscular volume (MCV) and (F) Mean corpuscular haemoglobin concentration (MCHC). (G) Gene expression of CD61 in Meg-01 from RNAseq Expressed as DESeq2 normalized readcounts. Data are expressed as the mean ± sd. Statistical difference was determined by Student’s t-test. **p < 0.01. [file Image_1.tif]
